# Assessment of knowledge and practice of venous thromboembolism (VTE) prophylaxis after cesarean section among gynecologists and obstetricians in Al-Najaf hospitals

**DOI:** 10.25122/jml-2021-0226

**Published:** 2021

**Authors:** Safa Emad Jawad Suker, Ayad Ali Hussein AL-meen, Ahmed Abduisahib Khawwam

**Affiliations:** 1. Department of Clinical Pharmacy, Al-Najaf Health Directorate, Al Najaf, Iraq; 2. Department of Clinical Pharmacy, Faculty of Pharmacy, University of Kufa, Al Najaf, Iraq; 3. Faculty of Dentistry, University of Kufa, Al Najaf, Iraq

**Keywords:** cesarean sections, venous thromboembolism, prophylaxis, guidelines

## Abstract

Venous Thromboembolism (VTE) is associated with high morbidity and mortality rates after cesarean sections. VTE is likely four-time greater following cesarean section than normal vaginal delivery. Despite a large number of published studies and the availability of well-evidenced guideline recommendations for VTE prevention, it is evident that these guidelines are poorly implemented with suboptimal use of a prophylactic thrombotic agent. The objective of our study was to assess the knowledge and practice of gynecologists and obstetricians about guidelines of VTE prophylaxis after cesarean section. An observational study included 57 gynecologists and obstetricians from all hospitals in Al-Najaf province. The study used a validated questionnaire consisting of 40 items where the correct response scored 1, giving an overall total score of 40. The total overall knowledge and practice score was calculated for participants, and the knowledge and practice levels were evaluated. Only 57 participants out of 67 completed the study giving a response rate of 85%. The mean overall score of practice and adherence was 0.51±0.09. This study showed inadequate practice towards VTE and poor adherence to prophylaxis guidelines because of many barriers, mainly the cost, poor patient adherence, and inconvenience to use guidelines in our patients.

## Introduction

In daily practice, gynecologists and obstetricians face many venous thromboembolism (VTE) cases; therefore, prophylaxis for this disorder is important before and after surgery [1]. Adherence to prophylaxis guidelines is essential. There are many standard guidelines concerning best practices for treating, diagnosing, preventing, and managing VTE like those published by the Royal College Of Gynecologists and Obstetricians RCOG [2], American College of Chest Physicians (ACCP) [3], American College of Obstetricians and Gynecologists (ACOG)[4], and Agency for Health Care Research and Quality (AHRQ)[5]. Moreover, Ward Thrombosis Day has an essentially educational purpose providing the family and the patient with enough information to advocate for VTE prevention, particularly in high-risk cases in the hospital [5]. Venous thromboembolism, which includes pulmonary embolism (PE) and deep venous thrombosis (DVT), is the formation of a clot in the venous system [6]. VTE is represented as the most important cause of morbidity and mortality in pregnant women after cesarean section [7]. The VTE represents the second direct cause of death, accounting for 13.8% of all maternal deaths in the world [8]. The most common risk factor for VTE is a cesarean section. The danger of VTE is four times greater following cesarean section than normal vaginal delivery [8]. Despite a large number of published studies and the availability of well-evidenced guideline recommendations for VTE prevention, it is evident that these guidelines are poorly implemented with suboptimal use of a prophylactic thrombotic agent. This study aims to assess the knowledge and practice of VTE prophylaxis after cesarean section in Najaf hospitals.

## Material and Methods

An observational study was conducted in all hospitals with gynecology and obstetrics wards in the center of Al-Najaf and the area outside the center. The hospitals included were Al-Zahraa Teaching Hospital, Al-Hakeem General Hospital, Al-Furat Middle Teaching Hospital, Al-Manathira General Hospital, Al-Haidarya General Hospital, and Al-Sajjad General Hospital in Najaf governorate, Iraq.

We used a validated questionnaire distributed to 57 gynecologists and obstetricians to assess the knowledge and practice of VTE prophylaxis after cesarean section in Najaf hospitals. Following this, we calculated the total overall knowledge and practice score for all participants.

## Results

**Figure 1.**
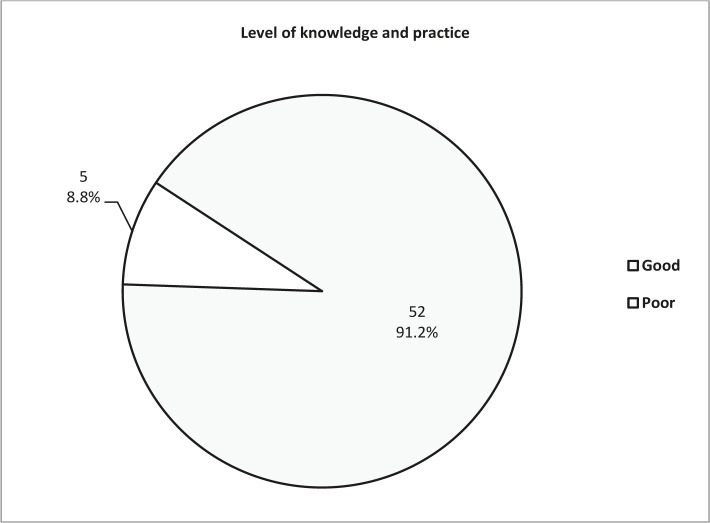
Proportional distribution of overall level of knowledge and practice of participant physicians.

Almost two-thirds of the participants were 40 years or older, with a mean age of 44.1±7.9 years. There were 25 participants holding board degrees (43.9%), the remaining participants had diploma degrees. Among the 57 participants, 12 (21.1%) had <5 years duration in clinical practice, 18 (31.6%) had a practice for 5–9 years, 14 (24.6%) for 10–14 years, and 13 (22.8%) had 15 years or more in practice, the mean duration in clinical practice was 10.7±7.5 years. Among the study participants, 36 physicians (63.2%) claimed they followed a specific guideline of thromboprophylaxis, and 21 (36.8%) did not, but they depend on their clinical practice and judgment (Table 1). Regarding the scores for different domains and overall level of knowledge and practice, the mean score ranged between 0.31 and 0.68 out of 1.0 for the five domains, with a higher mean score for the knowledge and practice regarding indications thromboprophylaxis followed by prescribing thromboprophylaxis agent (Table 2). However, the overall score was 0.51±0.09, reflecting inadequate (poor) knowledge and practice, where only 5 (8.8%) participants had a good level of knowledge and practice (Table 2 and Figure 1). No significant correlation was found between the knowledge and practice scores for all domains and the baseline characteristics of the studied group including, age, duration of practice, degree of specialty, and following specific guidelines, in all comparisons, P value >0.05 (Table 3). Moreover, the barriers of adherence and practicing thromboprophylaxis guidelines are summarized in Table 4, where the majority of participants claimed that they did not adhere to guidelines due to the cost of the thromboprophylaxis agents, followed by concern about bleeding risks, difficulty or inconvenience to use guidelines in our patients, and patients’ noncompliance.

**Table 1.** Baseline characteristics of the studied group (n=57).

**Variable**		**No.**	**%**
**Age (year)**	<40	18	31.6
	40–49	26	45.6
	≥50	13	22.8
**Duration in practice (Years)**	<5	12	21.1
	5–9	18	31.6
	10–14	14	24.6
	≥15	13	22.8
**Degree of specialty**	Board	25	43.9
	Diploma	32	56.1
Followed a guideline	Yes	36	63.2
	No	21	36.8

**Table 2.** Mean and standard deviations of knowledge and practice scores of participants for different domains.

**Domain (D)**	**Score**	
	**Mean**	**SD**
**D1: Knowledge and practice regarding general information about Thromboprophylaxis**	0.57	0.23
**D2: Knowledge and practice regarding Indications of Thromboprophylaxis**	0.68	0.11
**D3: Knowledge and practice regarding prescribing thromboprophylaxis agent**	0.65	0.12
**D4: Knowledge and practice regarding timing and dosing of thromboprophylaxis**	0.36	0.12
**D5: Knowledge and practice regarding scoring a risk factor**	0.31	0.12
**Overall Knowledge and practice score for all domains**	0.51	0.09

**Table 3.** Correlation of overall knowledge and practice score of participant physicians with other covariates.

**Correlations**	**Statistics**	**D1**	**D2**	**D3**	**D4**	**D5**	**Overall**
Hospital **	R	0.001	-0.137	-0.084	-0.135	-0.234	-0.141
	P value	0.996	0.309	0.533	0.318	0.286	0.302
Age (year) *	R	0.049	0.201	0.045	-0.269	-0.045	-0.014
	P value	0.716	0.133	0.739	0.043	0.739	0.918
Degree of Specialty **	R	-0.015	0.166	0.062	-0.231	-0.240	-0.120
	P value	0.911	0.217	0.645	0.084	0.072	0.375
Duration in clinical *practice as specialist	R	0.053	0.159	0.077	-0.204	-0.068	-0.005
	P value	0.697	0.237	0.567	0.128	0.613	0.972

R – correlation coefficient; * – Pearson's bivariate correlation analysis applied; ** – Spearman's bivariate correlation analysis applied.

**Table 4.** Barriers for poor practicing and adherence to thromboprophylaxis guidelines.

**Barrier**	**No.**	**%**
**High costs of thromboprophylaxis agents**	49	86.0
**Concern about bleeding risks**	41	71.9
**Difficult or inconvenient to use guidelines in our patients, and patients complain and noncompliance**	30	52.6
**Lack of awareness of guidelines**	28	49.1
**Need for new resources or facilities that are not available in our hospitals**	27	47.4
**Lack of familiarity with guidelines**	19	33.3
**Concern about infection resulting from wound hematomas**	14	24.6
**Lack of self-efficacy of some physicians (perceived inability to follow guidelines)**	14	24.6
**Disagreement between guidelines is confusing**	6	10.5
**VTE not practiced as a problem in our experience**	3	5.3

## Discussion

Venous thromboembolism is one of the most well-known life-threatening conditions contributing to a significant proportion of postpartum maternal death [9]. Women who deliver by CS are at high risk of VTE compared to those who deliver by normal vaginal delivery. Published epidemiologic data showed an almost twenty to eighty-fold increase in VTE incidence after CS [9]. Therefore, the current study aimed to assess the knowledge and practice of VTE prophylaxis after CS by gynecologists and obstetricians in Al-Najaf city. The total number of gynecologists and obstetricians in AL-Najaf city is 67 physicians, the available physicians in the hospital to answer the questionnaire is 62 physicians, and only 57 physicians accepted to participate in the study. The 85% response rate is a good response rate, above the minimum requirement to have good power of study [10–12]. Demographic characteristics of the participants revealed that the mean age was 44.1±7.9, and almost 2/3 of them were older than 40 and were practicing their specialty for a period (1–32 years). More than half of the participants have diploma degrees.

Regarding participants’ responses about whether they are following a specific guideline or not, 63.2% of them followed a specific guideline, while 36.8% did not follow any guideline and depended on their clinical practice and judgment. This finding is similar to a randomized clinical trial in Australia/New Zealand, which stated that most obstetricians depend on their clinical experience, practice, and judgment [13]. Another nationwide survey in all departments of obstetrics and gynecology in Germany showed that 19% of total respondents did not follow specific guidelines [8]. Nonetheless, the majority of those who practice guidelines followed the RCOG guideline, while others were least followed. Although the absolute VTE rate is low in the Iraqi population, no Iraqi national guideline is available, and the physicians depend on other available guidelines and their clinical practice experience. This result is similar to a cross-sectional study which revealed that patients receive prophylaxis according to RCOG by 85% compared to other guidelines where patients receive prophylaxis by 35% ACCP and 1% ACOG guideline [8].

The present study found that the knowledge and practice of guidelines regarding general information about thromboprophylaxis is 38.6%, the low score could be explained by an underestimation of the size of the problem and underutilization of VTE guidelines. Furthermore, regarding indications of thromboprophylaxis, the percent is 56.1%, with a mean score of 0.68±0.11. The practice and knowledge of the physicians included in the current study were lower than that recommended by different VTE thromboprophylaxis guidelines RCOG [2] and ACOG [4]. Similarly, what concerns prescribing thromboprophylaxis, there was poor practice and adherence to guidelines regarding prescriptions of the thromboprophylaxis agent, the correct practice and judgment of physicians was in 47.4% with a mean score of 0.65±0.12. However, it still needs further improvement in education [14–18]. Knowledge and practice of guidelines regarding timing and dosing are fundamental in any thromboprophylaxis guideline. Unfortunately, a large proportion of participants in the current study failed to address the correct timing and doses of thromboprophylaxis agents in their clinical practice before education. For instance, only 12.3% of participant physicians correctly judge the timing and doses for a non-obese patient with renal failure, and 26.3% correctly practice the thromboprophylaxis protocol in women older than 35 years undergoing cesarean section [19–20]. Because of the increasing anticoagulant response of Enoxaparin in a patient with renal failure (due to bioaccumulation of Enoxaparin leading to an increase in its side effect), Enoxaparin was assessed in previous clinical trials and documented with higher bleeding risk in such patients [21]. Therefore, adjustment of the dose is very important and highly recommended in those patients. Women with renal failure should receive a lower dose of thromboprophylaxis than women without renal failure [21]. Conversely, most of the participants in the current study did not respond correctly or were unaware of dose adjustment because they depend on other specialties, such as internal medicine physicians with subspecialty of renal disease who judge the dose for a patient with renal failure. The awareness regarding the dose of LMWH by assessing the patient according to RCOG guidelines will prevent the appearance of side effects [8, 15, 18]. Additionally, only 26.3% correctly responded to the timing and dosing of thromboprophylaxis for patients older than 35 years who need special care and specific thromboprophylaxis. Thus, there is a lack or poor practice regarding this item among participant physicians. Previous studies mention that women older than 35 years, with obesity, major surgery, and immobilization are greater risk factors and need special care and awareness regarding thromboprophylaxis and other diseases than thrombosis [15, 22]. Similarly, only 17.5% correctly score with a mean score of 0.31±0.12, and scoring the risk factors is the cornerstone of the thromboprophylaxis guideline. Good practice of scoring these risk factors significantly affects the choice of the prophylactic agent, the dose, and the type. Good scoring will improve the patient’s outcome, and literature confirms that scoring is very important [18, 23]. Unfortunately, the majority of participant physicians did not score the risk factor and did not assess the patient despite their knowledge of risk factors, which is maybe due to many challenges in daily practicing, particularly under the stressful environment in the labor ward. This problem has been identified in other countries such as Germany and India [8]. In general, for all these domains mentioned above, the overall practicing and adherence of participants is 8.8%, with a mean score of 0.51±0.09. However, there is still a need for further education targeting a mean score of one out of one with correct practice and adherence to these guidelines.

## Conclusions

This study showed inadequate practice towards VTE and poor adherence to prophylaxis guidelines because of many barriers, mainly the cost, poor patient adherence, and inconvenience to use guidelines in our patients.

## Acknowledgments

## Conflict of interest

The authors declare that there is no conflict of interest.

## Ethics approval

All ethical issues were approved by the Council of the Faculty of Pharmacy, the University of Kufa, and the Scientific Research (2405 in 25-11-2020) and Ethics Committee of the Al-Najaf Health Directorate (21528 in 24-9-2020).

## Consent to participate

Written and verbal consent was obtained from all participants. Data were collected following the World Medical Association Declaration of Helsinki 2013 for the ethical consideration of research that involves humans.

## Personal thanks

I wish to express my thanks to Professor Najah R. Hadi, Department of Pharmacology & Therapeutics, Faculty of Medicine, the University of Kufa for his help and support.

## Authorship

SEJS collected and analyzed data, AAHAm developed the hypothesis and AAK reviewed the findings and edited the manuscript.
